# Perspective: What Does Stunting Really Mean? A Critical Review of the Evidence

**DOI:** 10.1093/advances/nmy101

**Published:** 2019-02-25

**Authors:** Jef L Leroy, Edward A Frongillo

**Affiliations:** 1 Poverty, Health, and Nutrition Division, International Food Policy Research Institute, Washington, DC; 2 Department of Health Promotion, Education, and Behavior, University of South Carolina, Columbia, SC

**Keywords:** stunting, linear growth retardation, undernutrition, causality, marker, child development, earnings, chronic disease, birth outcomes, global development objective

## Abstract

The past decade has seen an unprecedented increase in attention to undernutrition, and drastically reducing child stunting has become a global development objective. The strong focus on linear growth retardation and stunting has enabled successful advocacy for nutrition, but with this focus has come some confusion and misunderstanding about the meaning of linear growth retardation and stunting among researchers, donors, and agencies active in nutrition. Motivated by the belief that a sharp focus will further accelerate progress in reducing undernutrition, we critically reviewed the evidence. The global attention to stunting is based on the premise that any intervention aimed at improving linear growth will subsequently lead to improvements in the correlates of linear growth retardation and stunting. Current evidence and understanding of mechanisms does not support this causal thinking, with 2 exceptions: linear growth retardation is a cause of difficult births and poor birth outcomes. Linear growth retardation is associated with (but does not cause) delayed child development, reduced earnings in adulthood, and chronic diseases. We thus propose distinguishing 2 distinctly different meanings of linear growth retardation and stunting. First, the association between linear growth retardation (or stunting) and other outcomes makes it a useful marker. Second, the causal links with difficult births and poor birth outcomes make linear growth retardation and stunting outcomes of intrinsic value. In many cases a focus on linear growth retardation and stunting is not necessary to improve the well-being of children; in many other cases, it is not sufficient to reach that goal; and for some outcomes, promoting linear growth is not the most cost-efficient strategy. We appeal to donors, program planners, and researchers to be specific in selecting nutrition outcomes and to target those outcomes directly.

## Introduction

Child undernutrition remains an important global health problem. Undernutrition increases susceptibility to illness and fatality, and if removed, 45% of child deaths would not occur. For surviving children, undernutrition has severe short-term (e.g., delayed cognitive development), medium-term (e.g., lower school achievement), and long-term consequences (e.g., lower earnings and higher probability of adult noncommunicable chronic diseases) (1).

The past decade has seen an unprecedented attention to undernutrition, as witnessed by examples of worldwide nutrition initiatives, global goal setting for nutrition, and high-level publications (2) (**Supplemental Text**). The goal of drastically reducing child stunting has taken center stage: the World Health Assembly's first global nutrition target is a 40% reduction by 2025 in the number of children <5 y old who are stunted (3).

The focus on linear growth retardation and stunting (see **Box 1** for definitions) has facilitated communication with policy makers, enabled successful advocacy for nutrition, and mobilized policy makers and donors to pay attention to undernutrition and its consequences. Rallying around stunting has contributed to garnering wide global support for nutrition which has been beneficial to the world. Building the strong and convincing stunting narrative, however, required leaving out important details about stunting's actual consequences. In this paper, we argue that along with the strong emphasis on linear growth retardation and stunting has come some confusion and misunderstanding about its meaning among researchers, donors, and agencies active in nutrition. Our paper is motivated by the concern that the current framing of stunting as the key global nutrition challenge has blurred our thinking. Not delivering on the ambitious stunting-reduction agenda may damage the current global nutrition momentum.

###

Box 1: **Linear growth retardation and stunting: what's the difference?**

**Linear growth retardation** (or linear growth faltering) is defined as a failure to reach one's linear growth potential. Linear growth retardation implies that (groups of) children are too short for their age, but does not imply that they are stunted (see below). As explained in the text, the number of children suffering from linear growth retardation is much higher than the number of children that are stunted.

**Stunting** is defined as having a height-for-age *z* score (HAZ) <–2SD. HAZ is calculated by subtracting an age- and sex-appropriate median value from a standard population and dividing by the SD of the standard population (4). The 2006 WHO growth standards are the recommended standard (5). In a healthy population, ∼2.5% of all children have a HAZ <–2SD. A higher percentage <–2SD is indicative of a deficient growth environment. Children who are stunted are a subset of those with linear growth retardation.

Our objective is to show that many outcomes commonly presented as consequences of linear growth retardation and stunting are not causally linked. We first illustrate how the nutrition community has emphasized the consequences of linear growth retardation and stunting and how this “causal” view has been widely adopted. Second, we critically review the scientific evidence linking linear growth retardation and stunting to other outcomes. Third, we recommend a fundamentally different evidence-based way of making use of linear growth retardation and stunting as measures of global development.

## What the Nutritional Science Community Is Telling the World about Linear Growth Retardation and Stunting

Linear growth retardation and stunting are associated with undesirable short-, medium-, and long-term outcomes in 5 domains: *1*) delayed child development (6), leading to lower school achievement and reduced earnings; *2*) reduced physical strength and work capacity (7), leading to reduced earnings; *3*) physiologic changes, contributing to adult noncommunicable diseases and increased mortality (8, 9); *4*) increased risk of cephalopelvic disproportion, leading to dystocia, mortality, and morbidity (1); and *5*) undesirable birth outcomes in the next generation (10), i.e., low birth weight or small-for-gestational-age (SGA) infants more likely to die or not grow adequately.

**FIGURE 1:**
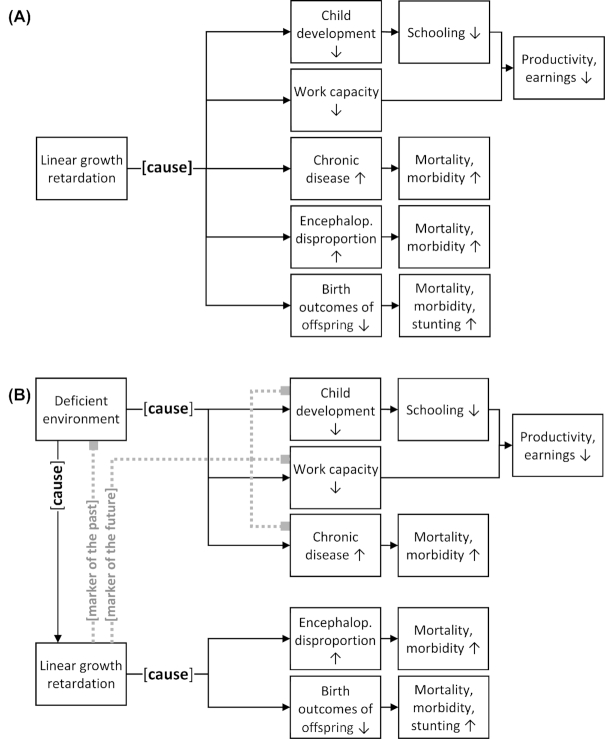
Commonly accepted framework showing the importance of linear growth retardation (A); and proposed framework distinguishing between child linear growth as an indicator reflective of the past, as indicator predicting the future, and as an outcome in its own right (B).

The scientific literature commonly presents these associations as being causal, i.e., claiming that linear growth retardation and stunting are a cause of the negative outcomes in these 5 domains (**Figure 1**A). A recent comprehensive literature review on the association between undernutrition in childhood and economic outcomes shows that this is a widely held view; over half of the 68 papers on linear growth or height made direct causal claims linking linear growth retardation (or stunting) to the 5 outcome domains (11).

If linear growth retardation (or stunting) is a cause of these negative outcomes, then it logically follows that improving child linear growth will improve these outcomes. The causal claims imply that any intervention aimed at improving linear growth will subsequently and automatically lead to improved outcomes in these 5 domains. We argue below that this causal evidence exists only for the last 2 domains.

This causal view is strongly embedded in the nutrition community. An informal survey of agencies and donors active in nutrition shows that they have generally adopted the view that linear growth retardation and stunting is a cause of developmental delays, lower levels of schooling, reduced earnings, and chronic disease risk. Eliminating linear growth retardation and stunting have become a primary development objective, based in part on believing that their elimination will lead to meaningful benefits in a large number of other domains. The causal thinking has also triggered research on, for example, aflatoxin and catch-up growth.

### Aflatoxin

The possible role of chronic exposure to aflatoxin (a mycotoxin produced by the fungus *Aspergillus* sp.) in the etiology of linear growth retardation and stunting is receiving increasing attention in the research and development community. The premise is that if aflatoxin exposure is a confirmed cause of linear growth retardation and stunting in children, then reducing aflatoxin exposure will ameliorate the negative consequences of poor growth (12–16). Since these consequences are limited (see subsequent sections), this motivation for researching the link between aflatoxin and linear growth is questionable. We add 2 nuances. First, effective aflatoxin control is important because aflatoxin is a group 1 carcinogen, and aflatoxin contamination of food crops impairs the ability of low- and middle-income countries to access export markets (16, 17). Second, aflatoxin (and mycotoxin) exposure may contribute to environmental enteric dysfunction, systemic inflammation, immunomodulation, and changes in the hepatic metabolism of micronutrients (14, 18). These short-term consequences (if confirmed) all warrant immediate preventive action. Furthermore, they all potentially limit young children's ability to fully develop into healthy and productive adults (19–21). Research should focus on these potential consequences of mycotoxin exposure, rather than on its contribution to linear growth retardation and stunting.

### Catch-up growth

Catch-up growth refers to accelerated growth that reduces a child's accumulated height deficit (22). Much recent work has reported catch-up growth in the absence of any nutrition intervention (23–28). Some studies reported an association between catch-up growth and child development, concluding that promoting growth during infancy and early childhood might contribute to better child development (25–28). These reports have received media attention (29). These conclusions are misleading for 2 reasons. First, much of the catch-up growth work has assumed that linear growth retardation and stunting negatively affect child (cognitive) development, and recovery from linear growth retardation or stunting is presented as if it will lead to improved cognitive outcomes (23–28). We show below that there is no evidence that linear growth and cognitive development are causally linked. Second, the recent studies use height-for-age *z* scores (HAZ), a measure that is statistically inappropriate to assess catch-up growth (22). In conclusion, none of these studies provided evidence of catch-up growth or determined that catch-up growth has long-term positive consequences on child development. Rather, they confirm that better linear growth is associated with better cognitive development, which is in line with existing knowledge (6). We add 2 caveats. First, the motivation for a much-cited study on catch-up growth is maternal short stature as a cause of poor birth outcomes (30), but the analyses used the statistically inappropriate HAZ (22). Second, recovery from child linear growth retardation or stunting may or may not be possible, but the reviewed studies do not provide evidence that it is.

## What Is the Evidence about Outcomes of Linear Growth Retardation and Stunting?

### Linear growth retardation and developmental delays

Linear growth retardation is associated with reduced cognition and motor development in middle- and low-income countries (6); the association between stunted growth and socioemotional development has received less attention (6). Linear growth retardation and poor development are associated through a set of shared determinants (suboptimal nutrition, inadequate care, and repeated infections). Based on current understanding, however, linear growth retardation is not part of the mechanistic path leading to delayed cognitive, motor, or socioemotional development (31). Two mechanisms have been raised as potentially causal. The first one is the hypothesized direct effect of smaller body size on reduced motor activity, which would limit the child's ability to explore and access stimulation (6, 32) and reduce opportunities for language, socioemotional, and cognitive development (33). Motor development, however, appears to be a consequence of factors including balance, myelination, muscle strength, and endurance, but not of body length (34). The second potential mechanism is the Rosenthal effect, whereby short child stature lowers caregivers’ expectations about children's developmental potential, which could then reduce the stimulation these children receive (31). This mechanism is not likely to be important in societies in which the majority of children suffer from some degree of linear growth retardation. In conclusion, there is no evidence that linear growth retardation (or stunting) causes delays in child development, and based on our current understanding of mechanisms, it is not likely that they are causally related.

### Linear growth retardation and earnings

In both developed economies and low-income settings, earnings are associated with height (7). Taller individuals have more schooling and better skills, which could explain the association, but the height-earnings association remains after controlling for cognitive and socioemotional capacity (7). There are several reasons to question the causality of this association. First, we could not find evidence for a credible biological (or other) mechanism that would explain the effect of stature on earnings at the population level. Second, the height-earnings association in developed economies indicates that relative height (rather than height in absolute terms) is of importance. The association will therefore not disappear when linear growth retardation is eliminated since that would not remove the distribution of heights at the population level. Third, with the use of longitudinal data from the Oriente study in Guatemala, Behrman et al. (35) statistically separated the effects of physical and intellectual human capital on wages, treating both types of human capital as statistically endogenous. We are not aware of other studies that used this method. In this population largely active in the agricultural sector, only intellectual and not physical human capital increased annual income (35). We conclude that a causal link between linear growth retardation (or stunting) and lower earnings is not supported by current evidence.

### Linear growth retardation and chronic diseases

Environmental influences during early development, such as poor nutrition, increase chronic disease risk later in life (36). Much early work on the developmental origins of disease focused on birth weight and infant size as measures of exposure (36, 37), which may have contributed to the belief that linear growth retardation and stunting are a cause of adult chronic disease risk (8, 9). Three interrelated categories of mechanisms underlying the effect of early environmental influences on chronic disease have been identified: changes in the structure and function of critical organs such as the brain, the pancreas, and the kidney; changes in gene expression; and changes in cellular senescence (37). Based on current knowledge, however, linear growth retardation and stunting are not part of the mechanistic path. Additionally, recent evidence from carefully conducted epidemiologic studies does not show an association between linear growth retardation (or stunting) and a number of chronic disease risk factors. Analyses of pooled data from 5 birth cohort studies in low- and middle-income countries (India, the Philippines, South Africa, Guatemala, and Brazil) showed that neither lower birthweight (birth length was not included) nor lower linear growth rates in the first 2 y of life were associated with increases in adult cardiovascular risk or plasma glucose concentration (38). A long-term follow-up of a South African cohort showed that children not stunted at 24 mo had a higher BMI-for-age *z* score (BMIZ) at 18 y than those who were stunted at 24 mo (39). Likewise, stunting at 12 mo of age in Peru was associated with a decreased risk of having a high BMIZ (40). We conclude that the evidence does not support a causal link between linear growth retardation (or stunting) and chronic disease.

### Linear growth retardation and encephalopelvic disproportion

Linear growth retardation at childhood reduces adult height. Shorter stature in women at adulthood, in turn, is associated with a higher risk of dystocia or difficult labor (1). Mechanical dystocia, or cephalopelvic disproportion, is a major cause of maternal and neonatal mortality and morbidity; the sequelae have important social, economic, and marital consequences (41). The association between maternal height and difficult labor is mediated by the size of the pelvic inlet; shorter women have a smaller pelvic inlet and are thus more likely to suffer from a mismatch between the size of fetal head and the dimensions of the birth canal (42, 43). Since both stature and pelvic size are linked to skeletal size, we can assume that the association between linear growth retardation at childhood and obstructed labor at adulthood is causal. Obstructed labor accounts for a small proportion (3%) of all maternal mortality or ∼10,000 deaths/y (44). Its disability burden is important (40% of the total number of years lost due to disability among all maternal disorders), but has dropped significantly over time (45). Which proportion of the mortality and disability burden could be averted by eliminating maternal short stature is not known. In conclusion, short stature and obstructed labor are causally linked, their mortality and morbidity burden is relatively small and declining, and the fraction of the mortality and morbidity burden attributable to linear growth retardation earlier in life is not known.

### Linear growth retardation and birth outcomes

A short mother (which could be due to linear growth retardation during her childhood) is more likely to have SGA children. This association is considered causal and due (in part) to maternal physical constraints associated with short stature (10). SGA children are at increased risk of neonatal and infant mortality and morbidity during the neonatal period and beyond (46). Being SGA is also responsible for up to 20% of stunting in children between the ages of 1 and 5 y (47). Maternal short stature is associated with an estimated 6 million SGA births in low- and middle-income countries annually (or ∼18.4% of the total) (44). Reducing SGA from its current prevalence of 19.3% in these countries to 10% would reduce neonatal deaths by 9.2% (or prevent 254,600 deaths) (48). Combining both estimates, eliminating SGA births that are due to maternal short stature would reduce neonatal deaths by an estimated 3.6% (or 97,200 deaths globally), a small proportion of the global total. Which proportion of the SGA-associated morbidity could be averted when eliminating maternal short stature is unknown. In conclusion, linear growth retardation at childhood is causally linked to an increased risk of giving birth to SGA children. Eliminating maternal short stature would have a modest effect on neonatal mortality and an unknown effect on child morbidity.

## Distinguishing between Linear Growth Retardation and Stunting as a Marker Compared with as an Outcome

Linear growth retardation and stunting are associated with—but based on available evidence do not cause—delayed child development, reduced earnings at adulthood, and chronic diseases. Linear growth retardation is a cause of difficult birth and poor birth outcomes. From these findings, we identify 2 distinct uses of linear growth retardation and stunting. First, the association between linear growth retardation (or stunting) and other outcomes makes it a useful marker. Second, the causal links with difficult birth and poor birth outcomes makes linear growth retardation and stunting outcomes of intrinsic value (Figure 1B). This marker compared with outcome distinction in relation to linear growth retardation and stunting has been made previously (49, 50).

### Linear growth retardation and stunting as markers reflective of past and predictive of future

Healthy linear growth requires children to consume adequate diets, to receive proper care, and to be healthy. These immediate determinants depend on food security, caregivers’ nutrition and health knowledge, and access to and proper use of health services (1). A change in the severity of linear growth retardation (or stunting) is indicative of changes in these immediate and underlying determinants. Linear growth retardation and stunting are markers of the inadequacy of the environment to which children have been exposed.

Since linear growth retardation and poor cognition share many of the same determinants (including suboptimal nutrition, inadequate care, and repeated infections), improvements in these determinants can be expected to improve both growth and cognition. Improved linear growth does not lead to improved cognition per se, but it can predict better cognition. Linear growth retardation and stunting in groups of children predict future poor school achievement and progress, lower cognition, reduced earnings, and a higher probability of living in poverty (51, 52).

Linear growth retardation and stunting often are used implicitly as markers of both the past and future. When a high stunting prevalence is reported for a region, 2 messages are implied. First, children grow up in a deficient growth environment. Second, as a consequence of growing up in this environment, they are unlikely to realize their full developmental and economic potential in the future.

### Linear growth retardation and stunting as outcomes of intrinsic value

Linear growth retardation is causally linked to difficult child birth and poor birth outcomes. Linear growth retardation is therefore an outcome of intrinsic value, since a reduction in linear growth retardation (or stunting) is expected to directly improve these outcomes. Linear growth here is part of the mechanistic path and not just a marker of other outcomes (Figure 1B).

## Just Semantics?

A more careful distinction between linear growth retardation (or stunting) as a marker compared with an outcome has a number of practical implications.

### Improving linear growth is often not necessary

Interventions may positively and meaningfully affect important nutrition outcomes without providing the dose or inputs necessary to improve linear growth. That is, for many nutrition outcomes (e.g., infant and young child feeding practices, dietary adequacy, and micronutrient status), nutrition interventions will have positive, meaningful, and observable effects before linear growth improves. For example, a combination of interpersonal counseling, a national mass media campaign, and community mobilization in Vietnam and Bangladesh successfully improved complementary feeding practices, but not linear growth (53, 54). Impacts on linear growth retardation or stunting possibly required larger improvements in feeding practices or improvements in other determinants such as health. Furthermore, equating lack of impact on linear growth to program failure discounts the importance of other outcomes and interventions to improve them. Finally, several nutrition interventions are highly effective at improving children's well-being but have no effect on linear growth. Optimal breastfeeding and vitamin A supplementation, for instance, reduce morbidity and mortality but do not improve linear growth (1).

### Improving linear growth is not sufficient

Eliminating linear growth retardation is not sufficient to ensure children develop to their full potential. Children who grow adequately, but who lack adequate stimulation at home or attend poor-quality preschool and primary education, are unlikely to fully develop.

### Improving linear growth may not efficiently address other outcomes

Addressing outcomes associated with linear growth retardation or stunting directly is likely more efficient than addressing these outcomes indirectly through linear growth. The effect size of nutrition interventions on cognitive outcomes is an estimated 4–5 times smaller than that of interventions providing stimulation (31). Other examples include addressing the problems of obstructed labor and SGA for which other strategies are more efficient than reducing linear growth retardation (Supplemental Text).

### Eliminating fatalism

The observation that the first 2 y of life are the period of most rapid growth failure (55) and interventions beyond this age have little or no impact on child linear growth (56) have led to a view that interventions outside this window are unlikely to have meaningful effects (30). Linear growth retardation continues beyond the first 1000 d (57), however, and the biological window of opportunity for improving linear growth does not necessarily coincide with windows for other outcomes. Regions in the brain responsible for higher cognition (e.g., reasoning, problem solving) have a maturational course that extends into adolescence (58). The focus on the first 1000 d should be maintained, but nutrition, health, and development efforts need to extend beyond this period. Current evidence does not provide good guidance on which interventions to implement after 2 y of age or what improvements in which domains could be expected. Research is needed to assess the potential to improve nutritional status beyond 2 y of age (59), to test the impact of different packages of interventions on undernutrition and its functional consequences, and to identify optimal timing for improving these outcomes cost effectively, without increasing chronic disease risk.

### Getting other sectors on board for nutrition-sensitive interventions

Solving the world's nutrition problems will require both nutrition-specific and nutrition-sensitive interventions (60). Nutrition-sensitive interventions both address the underlying causes of undernutrition (e.g., poverty and food insecurity) and incorporate specific nutrition goals and actions. The narrow focus on linear growth as a nutrition outcome, however, may create a barrier for other sectors to engage. Nutrition-sensitive agriculture programs, for instance, can contribute to improving access to and consumption of high-quality diets, but these programs cannot alone improve linear growth (61). Likewise, nutrition-sensitive social protection can reduce poverty and improve food security, but should not be expected to directly improve child growth.

## Proposed Way Forward

### The need for specificity

Donors, program planners, and researchers in nutrition should be specific in using terminology and avoid using undernutrition and linear growth retardation (or stunting) as synonyms, as is often done. Many forms of undernutrition are biologically unrelated to linear growth retardation and stunting, and linear growth retardation and stunting are not merely a consequence of nutritional inadequacy. Linear growth retardation and stunting are not synonyms (Box 1). Donors, program planners, and researchers should be explicit about reasons for focusing on linear growth: is it used for population assessment, to count those affected, or in program design and evaluation? In programs, is it used as a marker of another outcome (and why is that outcome not addressed directly) or is it an outcome of immediate interest (and why was it chosen as an objective)?

### Population assessment

Because linear growth retardation and stunting mark the inadequacy of the environment to which children have been exposed, they provide a good indicator for population assessment. The severity of linear growth retardation and stunting in groups of children can be used to compare countries or regions within a country, and can be used to monitor progress of children of the same age distribution over time.

### Counting cases

The use of stunting (defined as an HAZ <–2SD) to count the number of children affected has inherent limitations (62). First, there is no biological or clinical basis for the arbitrary cut-off; nothing changes just above or below –2SD. Second, the number of stunted children vastly underestimates the number of children who are affected by an inadequate growth environment, as the entire HAZ distribution is shifted in populations with a prevalence of stunting >2.5% (62, 4). In Burundi, for instance, 65% of children between 24 and 42 mo of age were counted as stunted (63). The entire HAZ distribution was shifted to the left, which implies that a much larger percentage, if not all children, suffered from a deficient growth environment. Moreover, estimates that use stunting to count those affected will be inaccurate. For instance, estimating the cost per case of stunting averted assigns all program costs to the (few) children who crossed the cut-off and ignores the benefits incurred by others, thus underestimating impact and inflating costs relative to effectiveness. Nevertheless, relative differences in stunting prevalence are useful for population assessment, e.g., to compare countries or changes in populations with the same age distribution over time.

### Programs, interventions, and impact evaluation

Although relatively easy to assess, linear growth retardation and stunting should not be a primary outcome for the purposes of evaluating programs and interventions. Linear growth retardation and stunting are causally linked to only 2 negative outcomes which can be more effectively addressed through direct interventions. Donors and implementers should select primary outcomes that are directly relevant, such as early childhood development, dietary adequacy, nutrient status, and health, thereby eliminating the risk of reducing a program's success to its ability to improve linear growth. Assessing outcomes such as early childhood development, dietary adequacy, and nutrition status are currently more difficult and costly than measuring child length or height. A wider use of these outcomes may, however, spark investments in the development of more field-friendly measures. Assessing linear growth as a secondary outcome might be useful to evaluate if a program was successful in improving the full set of conditions necessary for linear growth.

## Conclusions

The current global attention to undernutrition provides an unprecedented opportunity to improve the well-being of billions of people, with positive consequences for their health, development, schooling, and earnings. Rallying around linear growth retardation and stunting has resulted in extraordinary nutrition momentum, but a narrow focus on these outcomes could have important downsides going forward. Equating lack of impact on linear growth retardation or stunting to program failure unnecessarily discounts other important outcomes and interventions to improve these conditions. In many cases a focus on linear growth retardation and stunting is not necessary to improve the well-being of children; in many other cases, it is not sufficient to reach that goal; and for some outcomes, promoting linear growth is not the most cost-efficient strategy. To maintain global nutrition momentum, a sharp focus of nutrition investments, policies, and programs on outcomes that truly matter will help accelerate progress towards the well-being of children in disadvantaged communities.

## Notes

Perspective articles allow authors to take a position on a topic of current major importance or controversy in the field of nutrition. As such, these articles could include statements based on author opinions or point of view. Opinions expressed in Perspective articles are those of the author and are not attributable to the funder(s) or the sponsor(s) or the publisher, Editor, or Editorial Board of *Advances in Nutrition*. Individuals with different positions on the topic of a Perspective are invited to submit their comments in the form of a Perspectives article or in a Letter to the Editor.

This research was supported by the CGIAR Research Program on Agriculture for Nutrition and Health (A4NH) led by the International Food Policy Research Institute. These ideas were presented at the 2017 International Congress of Nutrition in Buenos Aires in a symposium partially supported by the Bill & Melinda Gates Foundation, through Alive & Thrive, managed by FHI 360.

Supplemental Text is available from the “Supplementary data” link in the online posting of the article and from the same link in the online table of contents at https://academic.oup.com/advances/.

Abbreviations used: BMIZ, BMI-for-age *z* score; HAZ, height-for-age *z* score; SGA, small for gestational age.
